# Multidirectional facets of obesity management in the metabolic syndrome population after liver transplantation

**DOI:** 10.1002/iid3.538

**Published:** 2021-10-01

**Authors:** Kinga Czarnecka, Paulina Czarnecka, Olga Tronina, Teresa Bączkowska, Magdalena Durlik

**Affiliations:** ^1^ Department of Transplant Medicine, Nephrology and Internal Diseases Medical University of Warsa Warsaw Poland

**Keywords:** gastrointestinal microbiome, liver transplant, metabolic syndrome, nonalcoholic fatty liver disease, obesity management, visceral obesity

## Abstract

The obesity pandemic has resulted in an increasing demand for liver transplantation and has significantly altered the profile of liver transplant candidates in addition to affecting posttransplantation outcomes. In this review, we discuss a broad range of clinical approaches that warrant attention to provide comprehensive and patient‐centred medical care to liver transplant recipients, and to be prepared to confront the rapidly changing clinical challenges and ensuing dilemmas. Adipose tissue is a complex and metabolically active organ. Visceral fat deposition is a key predictor of overall obesity‐related morbidity and mortality. Limited pharmacological options are available for the treatment of obesity in the liver transplant population. Bariatric surgery may be an alternative in eligible patients. The rapidly increasing prevalence of nonalcoholic fatty liver disease (NAFLD) is a global concern; NAFLD affects both pre‐ and posttransplantation outcomes. Numerous studies have investigated pharmacological and nonpharmacological management of NAFLD and some of these have shown promising results. Liver transplant recipients are constantly exposed to numerous factors that result in intestinal microbiota alterations, which were linked to the development of obesity, diabetes type 2, metabolic syndrome (MS), NAFLD, and hepatocellular cancer. Microbiota modifications with probiotics and prebiotics bring gratifying results in the management of metabolic complications. Fecal microbiota transplantation (FMT) is successfully performed in many medical indications. However, the safety and efficacy profiles of FMT in immunocompromised patients remain unclear. Obesity together with immunosuppressive treatment, may affect the pharmacokinetic and/or pharmacodynamic properties of coadministered medications. Individualized immunosuppressive regimens are recommended following liver transplantation to address possible metabolic concerns. Effective and comprehensive management of metabolic complications is shown to yield multiple beneficial results in the liver transplant population and may bring gratifying results in improving long‐term survival rates.

## Graphical abstract

The obesity pandemic has resulted in an increasing demand for liver transplantation and has significantly altered the profile of liver transplant candidates in addition to affecting posttransplantation outcomes. Metabolic syndrome following liver transplant is a common phenomenon and is implicated as an important contributor to decades‐long unimproved long‐term outcomes after organ transplant procedure. In this review, we discuss a broad range of clinical approaches that warrant attention to provide comprehensive and patient‐centred medical care to liver transplant recipients, and to be prepared to confront the rapidly changing clinical challenges and ensuing dilemmas.

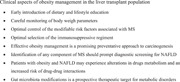

## 1 INTRODUCTION

Metabolic syndrome (MS) is one of the most challenging global health concerns; the increasing prevalence of MS and its complications has reached epidemic levels in the general population, and this upward trend is projected to continue secondary to the significant increase in the global burden of obesity and type 2 diabetes mellitus (DM2).^1^ The obesity pandemic has resulted in an increasing demand for liver transplantation and has also significantly altered the profile of liver transplant candidates, in addition to affecting posttransplantation outcomes. Metabolic complications are commonly observed in patients after liver transplant and are implicated as a well‐defined risk factor for increased morbidity and mortality rates, as well as an important contributor to decades‐long unimproved long‐term outcomes following liver transplant procedure. The association between MS and numerous comorbidities has been long established. MS together with immunosuppressive therapy is the main contributor to posttransplantation cardiovascular (CV) morbidity, which accounts for 19%–42% of nongraft‐related fatal outcomes.^2^ Furthermore, cardiovascular diseases (CVDs) represent the third leading cause of death after liver‐related causes and malignancies, 1‐year posttransplantation.^3^ Metabolic disturbances have also been implicated in the rapid progression of organ fibrosis in liver transplant recipients.^4^, ^5^, ^6^, ^7^ Observational studies have confirmed that obesity and DM2 serve as independent risk factors for hepatocellular cancer (HCC).^8^, ^9^, ^10^

MS is defined as a combination of coexisting metabolic derangements, including abdominal obesity, hyperglycemia, dyslipidemia, and hypertension. However, numerous definitions of MS have been proposed over the years.^11^, ^12^, ^13^, ^14^ Various studies have defined obesity based on diverse anthropometric measures. Therefore, it is difficult to draw definitive conclusions, and establishing a standardized therapeutic strategy for the management of metabolic complications, particularly after liver transplantation is challenging. It is important to fill the knowledge gaps in our current understanding of obesity to be prepared to successfully manage the rapidly changing clinical challenges and ensuing dilemmas through a proactive approach that includes early diagnosis and prompt treatment of the modifiable metabolic risk factors (Table 1).

**Table 1:** Clinical aspects that warrant consideration in the liver transplant population with metabolic complications

Clinical aspects of obesity management in the liver transplant population	
Early introduction of dietary and lifestyle education Careful monitoring of body weight parameters Optimal control of the modifiable risk factors associated with metabolic syndrome (MS) Optimal selection of the immunosuppressive regiment Effective obesity management is a promising preventative approach to carcinogenesis Identification of any component of MS should prompt diagnostic screening for nonalcoholic fatty liver disease (NAFLD) Patients with obesity and NAFLD may experience alterations in drugs metabolism and an increased risk of drug–drug interactions Gut microbiota modifications is a prospective therapeutic target for metabolic disorders	

## 2 MS

Numerous modifiable and nonmodifiable predisposing factors contribute to the multifactorial etiology of MS (Table 2). Patients awaiting liver transplantation and those who already underwent this procedure constitute a specific population of patients with MS. Multiple confounders observed in the pretransplant period preclude MS diagnosis. Vasodilatation and a reduced effective circulating volume, which is a known consequence of portal hypertension, result in low blood pressure. Significantly impaired hepatic synthetic function leads to underreported serum glucose and lipid levels. Ascites may affect accurate evaluation of obesity and waist circumference (WC),^25^ all of which imply that some preexisting risk factors promoting MS development may become apparent only after liver transplantation. Therefore, the prevalence of MS in the pretransplant period is poorly characterized and may vary from 5.4% to 22% depending on the study.^15^, ^26^ Additionally, organ transplant recipients are exposed to a wide variety of transplant‐specific risk factors for MS that differ from those observed in the general population. Many of these are nonmodifiable; therefore, it is of paramount importance to identify the potential areas of intervention based on modifiable risk factors of MS (Table 2).

**Table 2:** Modifiable and nonmodifiable factors associated with the metabolic syndrome development after liver transplantation

Modifiable factors	Nonmodifiable factors
Weight^2^ Body mass index (BMI)^15^, ^18^, ^19^, ^20^, ^21^, ^22^, ^23^, ^24^ Change in BMI^18^ Triglycerides^15^ High‐density lipoprotein^15^ Nonalcoholic steatohepatitis^21^ Hypertension^21^, ^22^	Age^2^, ^15^, ^16^, ^17^ Alcoholic cirrhosis^2^ Hepatitis C cirrhosis^2^ Cryptogenic cirrhosis^2^, ^15^

The literature findings suggest that MS in the posttransplant period may develop in 44%–58% of individuals, which makes it one of the most common complications following liver transplant and one that distinctly exceeds the prevalence estimates for the general population.^1^, ^2^, ^4^, ^15^, ^18^, ^25^, ^26^ A recent meta‐analysis reported MS in 39% of liver transplant recipients, with new‐onset MS diagnosed in 35% of patients.^20^ The posttransplant prevalence of MS may be significantly underestimated owing to the limited number of studies conducted on the subject, and also owing to the disparate definitions of MS used across different studies.

Therefore, optimal control of the modifiable risk factors associated with MS is important to improve long‐term outcomes after liver transplantation.

## 3 OBESITY

Studies have reported that 15%–30% of patients with initially normal body weight are diagnosed with obesity during the first year after liver transplant, this rate was shown to reach over 40% in 3‐year observation.^26^, ^27^, ^28^, ^29^ Patients with pretransplant obesity tend to remain overweight thereafter.^18^, ^30^ Posttransplantation weight gain was shown to increase significantly over 6 months after liver transplant and, more importantly, it has a well‐documented relationship with an increased risk of MS development and its complications.^27^ Therefore, close and careful clinical monitoring is essential during the early posttransplantation phase.

A growing body of evidence confirms the prominent role of body fat distribution, rather than overall body fat content, in increasing cardiometabolic complications risk. Numerous studies point to the significant structural and functional differences between visceral and subcutaneous adipose tissue depots to be of paramount importance.^31^, ^32^, ^33^, ^34^ Visceral adipose tissue, as a metabolically active organ, impacts cardiometabolic risk and is independently associated with malignancies.^35^, ^36^, ^37^, ^38^, ^39^ Scientific findings determined that HCC in patients with cirrhosis and recurrent HCC after liver transplantation are attributable to high visceral fat deposition.^40^ Published literature sources offer evidence for the existence of strong correlation between obesity and both incidence and mortality attributable to other cancers. It is estimated that an increase in the body mass index (BMI) by 5 kg/m^2^ may augment cancer‐related mortality by 10%.^41^ Corresponding conclusions were drawn based on the surgically induced weight loss.^42^, ^43^ These findings suggest that effective obesity management may represent a promising preventative approach to carcinogenesis.

A review of available literature provides conflicting information on the influence of pretransplant obesity on the posttransplant setting.^44^, ^45^, ^46^, ^47^, ^48^, ^49^ Although individuals with obesity may benefit from the organ transplantation procedure in terms of reducing waiting‐list‐mortality, it is equally indisputable that liver transplantation in patients with obesity prolongs the operation time, overall hospital and ICU stay, increases the risk of infectious and hemorrhagic complications and consequently the risk of CV events and malignancies, which cumulatively reduce patient and graft survival.^44^, ^50^, ^51^, ^52^, ^53^, ^54^

The broad variations of the studies' outcomes may be attributable to disparate obesity definitions applied in the studies, the time of observation, and survival rate adjustment for other comorbidities, particularly diabetes mellitus (DM), which appears to have a major impact.^47^ Obesity, in a substantial number of studies, was defined by BMI values, which in patients with end‐stage liver disease have several clinical limitations and show poor correlation with the posttransplant outcomes.^55^ Moreover, BMI values, which reflect overall fat content in the human body, poorly correlate with body fat distribution, especially with abdominal obesity, which is considered a better predictor of overall obesity‐related morbidity and mortality.^56^, ^57^, ^58^ Of note, sarcopenia, which coincides in 40%–70% of patients with cirrhosis, has not been taken into consideration in any of the studies.^59^, ^60^ Therefore, most recent research suggests to use visceral fat area in the identification of individuals with obesity among this specific population.^61^

Waist‐to‐hip ratio (WHR) is a simple and convenient tool to assess regional fat distribution. In several studies, WHR was demonstrated to be predictive of diabetes and CV events.^62^, ^63^ Regretfully, WHR measurement was only moderately useful as a measure of visceral adiposity.^64^ WC surpasses WHR in visceral fat assessment, hence, better mirrors metabolic disturbances and CV risk.^65^ Moreover, as evidenced by Sigit et al. abdominal obesity assessed by WC values, rather than overall obesity assessed by BMI, showed strong association with the occurrence of MS.^31^

### 3.1 Obesity management

Dietary restrictions and physical activity are considered the cornerstone of obesity management. Effective weight‐reduction strategies are shown to yield multiple beneficial results. Weight reduction of up to 5% of the initial body weight alleviates liver steatosis and up to 7% may result in recovery of nonalcoholic steatohepatitis (NASH). Further weight loss of at least 10% may benefit liver fibrosis.^66^, ^67^ Unfortunately, many liver transplant recipients are known to insufficiently respond to this approach.^16^, ^66^

### 3.2 Pharmacological management

Few pharmacological options are available for the treatment of obesity in the liver transplant population owing to the limited effectiveness and considerable adverse effects. Orlistat, a reversible inhibitor of pancreatic lipase, showed promising results in the pharmacological management of obesity in the general population, and the same effect was hoped for liver recipients.^68^, ^69^ Several studies determined a presumptive beneficial influence of orlistat in improving insulin resistance and lipid profile in individuals with obesity, which prompted further investigation of this medicine as a therapeutic option in nonalcoholic fatty liver disease (NAFLD) and NASH.^68^, ^69^ However, to date, scientific data are sparse in this regard. Some authors have reported orlistat‐related improvement in liver fibrosis and inflammation in patients with NASH and reduction of steatosis in patients with NAFLD.^70^, ^71^ On the other hand, most recent meta‐analysis did not confirm orlistat‐related beneficial effects on liver histology in NAFLD and NASH.^72^ These findings are in line with a randomized study by Harisson et al. which did not confirm orlistat's advantageous impact either on liver steatosis nor on metabolic derangements.^73^

Based on current knowledge, orlistat should be used with caution in liver transplant recipients as its mechanism of action interferes with the process of gastrointestinal digestion and absorption, hence, may significantly affect the serum levels of immunosuppressive agents. There are well‐documented clinically relevant interactions between orlistat and cyclosporin resulting in reduced bioavailability of the latter.^74^, ^75^ On the other hand, Cassiman et al. proved short‐term safety of orlistat administration in the liver transplant population with a tacrolimus‐based immunosuppression regimen, provided that serum levels of immunosuppressants and dietary restrictions were strictly monitored and obeyed. The study performed by Cassiman et al. did not include a formal control group; therefore, the efficacy of orlistat is unfeasible to assess based on this study.^76^

In view of vitamin E's well‐established antioxidative and antiinflammatory properties, its supplementation appears to be a promising therapeutic option for obesity and MS.^77^ Recent scientific reports evidenced additional antiobesity, antidiabetic, antihypertensive, and antihypercholesterolaemic effects of vitamin E.^78^, ^79^, ^80^, ^81^, ^82^ A cross‐sectional study performed by Aasheim et al. documented that individuals with morbid obesity, regardless of sex, have considerably lower serum levels of vitamin E, which absorption was determined to be impaired in MS subjects in comparison with healthy controls in Mah et al. study.^83^, ^84^ High inflammatory response and oxidative stress were proposed as the main incriminating factors responsible for this phenomenon. Nevertheless, limited data are available to confirm the direct association between vitamin E and obesity or MS. Concerns regarding the safety profile of long‐term administration of vitamin E might be a limiting factor.^85^, ^86^, ^87^

### 3.3 Bariatric surgery

Bariatric surgery (BS) appears to be a feasible and safe, thought challenging, procedure to be performed in the transplant population. It is universally perceived as an alternative treatment for patients with morbid obesity, who failed to respond to noninvasive therapeutic methods. A growing body of evidence indicates that in addition to satisfactory surgically induced weight loss, BS is associated with significant improvement in patients' metabolic profiles with favorable changes in liver histology. However, to obtain gratifying results with the minimum risk for the patient, there are certain points to consider during qualification of potential candidates such as deliberate choice of surgical technique combined with close monitoring of perioperative complications, regular verification of serum levels of immunosuppressive drugs, adequate supplementation of microelements and vitamins.

A lot of controversies used to surround bariatric procedures performed in the posttransplant setting, while currently what poses an even more debatable matter are the advisable, safe time frames between the transplant procedure and BS. Surgical interference in the gastrointestinal tract poses a risk for postoperative complications, impaired absorption of immunosuppressants, and unnecessary modifications in antirejection treatment. An interval of at least 1 year between liver transplantation and BS has been suggested to minimize the risk of plausible complications. However, no guidelines have been developed so far.^61^

The best‐investigated BS method in the liver transplant population is laparoscopic sleeve gastrectomy (LSG). However, successful cases of Roux‐en‐Y gastric bypass (RYGB) and intragastric balloon have been reported. Although RYGB gives faster results in weight reduction and a potentially faster resolution of obesity‐related complications, nutritional deficiencies after RYGB resulting from malabsorption may make this procedure less attractive for liver transplant patients. LSG is considered not to interfere with tacrolimus or mycophenolic acid (MPA) therapy; therefore, antirejection therapy modifications are not required.^88^, ^89^ However, inconsistent data are available regarding surgery‐induced weight loss and postoperative complications after LSG.^88^, ^90^ Tsamalaidze et al. in the retrospective case‐control study showed comparable operative‐time, postoperative 90‐day morbidity outcomes, and similar postoperative BMI changes between the obese population after liver transplant and individuals with obesity from the general population. Nevertheless, with regard to surgery‐related excess bodyweight reduction, a significant advantage was observed in the group with obesity from the general population at 2‐year follow‐up.^88^ In contrast, a meta‐analysis of Lazzati et al. reported satisfactory weight loss of 66% following LSG in liver recipients at 2‐year follow‐up, which was consistent with the results obtained in the general population. Posttransplantation morbidity and mortality rates documented in the meta‐analysis were higher but acceptable.^90^ However, another study documented a significant number of reoperations, reaching 33%.^91^

Research conducted on individuals with obesity in the general population suggests a statistically significant advantage of laparoscopic gastric banding (LGB) in weight loss reduction compared with LSG.^92^ Unfortunately, given that gastric banding requires implantation of foreign body, it may be of limited utilization in the transplant population owing to the plausible increased risk of infection development and more demanding technical issues.

In addition to its proven efficacy in patients with obesity, BS‐induced DM and NASH remission have been reported in patients after organ transplantation following LSG.^88^ One of the studies reported a simultaneous decrease in the MS prevalence from 70% to 14% in the general population.^93^ Duchini et al. observed that RYBG performed in liver transplant recipients with morbid obesity and a medical history of recurrent NASH was associated with a significant histological improvement in liver steatosis and complete resolution of liver fibrosis. The beneficial effects of surgically induced weight loss led to normalization of lipid and glucose parameters. Interestingly, in contrast to many other studies, the authors did not report any interference with the postoperative pharmacokinetics (PK) of immunosuppressants.^94^, ^95^

Despite sparse data regarding the role of pretransplant obesity on posttransplant outcomes, cumulative analysis suggests that pretransplantation obesity adversely affects patient and graft survival. Dietary changes and physical activity are still considered first‐line treatment of obesity, which regretfully, was evidenced to be ineffective in many liver transplant recipients. Research has shown that BS represents a safe and feasible alternative in patients diagnosed with morbid obesity. Emerging scientific findings postulate that effective obesity management may prevent carcinogenesis. Visceral fat area estimation may serve as a promising diagnostic tool for efficient identification of patients at high risk of metabolic complications among the liver transplant population, which has a chance to be introduced into routine practice following the availability of more data in the subject.

## 4 NAFLD

NAFLD is considered a hepatic manifestation of MS. To date, NAFLD is ranked as the third most common indication for liver transplantation.^96^, ^97^ However, due to the global expansion of obesity and diabetes, it is also the fastest growing indication for the procedure.^47^ Data regarding the prevalence of NASH in liver transplant candidates is sparse and accounts for 9.7%–47.5%.^96^, ^98^, ^99^ Both NAFLD and NASH exert a significant recurrence rate in the transplanted organ and are proven to adversely influence other coexisting metabolic derangements.^99^

NAFLD is a general medical term encompassing nonalcoholic fatty liver (NFL‐steatosis) and NASH. Steatosis is defined as the accumulation of triglycerides in >5% of hepatic cells. NASH is the most advanced and aggressive form of NAFLD and may predispose to liver fibrosis and HCC development.^100^ Histological findings in NASH show inflammatory cells infiltrations and hepatocellular ballooning in addition to simple steatosis. NAFLD in the posttransplant setting may be a consequence of either the recurrence of the disease or its de novo development. The prevalence estimates for de novo cases are accounted for 18%–33%.^101^, ^102^, ^103^ Nevertheless, the accurate scope of the phenomenon is undeterminable, due to the limited studies conducted on the subject as well as the significant number of underreported cases of the disease in the pretransplant period.^99^ Existing data indicate that NAFLD development in the allograft may occur in 100% of individuals transplanted for NASH in 5‐year observation.^101^ There are available studies indicating significant distinguishing features between de novo development of the disease and its recurrence with meaningful clinical implications. Recurrent NAFLD in the transplanted liver is presumed to present a more severe course and may be an irreversible process.^103^, ^104^, ^105^

In view of no transplant‐specific guidelines for NAFLD management, recommendations for the general population are applied. In accordance with the EASL‐EASD‐EASO Clinical Practice Guidelines, the presence of insulin resistance or any other component of MS should prompt diagnostic screening for NAFLD and, conversely, a diagnosis of NAFLD should lead to proactive search of all MS constituents.^97^ Notably, approximately 7% of individuals with normal body weight and unimpaired levels of liver enzymes may be NAFLD affected.^106^

In addition to obesity and DM2, NAFLD is considered a risk factor of HCC. As evidenced by one of the most recent studies conducted, nearly 12% of HCC cases may occur in noncirrhotic patients with NAFLD being the most frequently reported underlying liver disease.^107^ However, currently no specific recommendations are available for HCC screening in patients with NAFLD. The list of NAFLD‐related comorbidities lengthens as more studies in the subject are being released.

### 4.1 Management of NAFLD

Dietary restrictions and lifestyle modifications are the mainstay of NAFLD therapy. In NAFLD patients with obesity/overweight reduction in initial body weight by at least 7% resulted in a histological liver improvement.^108^

To date, numerous pharmacological interventions have been investigated in the subject. However, the obtained results were not conclusive. The most promising outcomes were obtained with thiazolidinediones. Several randomized clinical trials (RCTs) conducted on the general population determined pioglitazone effect in improving liver histology and even complete resolution of NASH.^97^, ^109^, ^110^ Of note, interventions with other thiazolidinedione group representatives—rosiglitazone and troglitazone—did not confirm the results.^97^, ^111^, ^112^ Despite the apparent safety and effectiveness of pioglitazone in NASH management, significant drug‐related adverse effects have been reported, most commonly weight gain and peripheral edema.^109^, ^110^ However, literature findings also mention an increased risk of bone fractures, particularly in female subjects.^113^ Long‐term adverse effects imposing the discontinuation of treatment limit the actual impact of these medicines on the progression of liver fibrosis. Initial studies that investigated the use of incretin mimetics, a new class of antidiabetic drugs, showed beneficial effects in patients with NASH. However, further research is required to conclusively establish the role of incretin mimetics before these drugs can safely be introduced into therapy guidelines.^97^, ^103^, ^114^ Data on vitamin E administration is sparse. There are available studies determining the effectiveness of the medicine in improving NASH by 36% in comparison with placebo.^97^, ^115^ However, this finding is refuted by other authors who observed no improvement following vitamin E therapy. In addition to its questionable effectiveness, the safety of long‐term vitamin E administration remains controversial; increased overall mortality, hemorrhagic stroke, and prostate cancer have been reported following vitamin E therapy.^85^, ^86^, ^87^ Prospective results of resmetirom have been reported in Phase 2 of clinical trials in NAFLD treatment in the general population. However, observed gastrointestinal disorders may, theoretically, significantly interfere with the bioavailability of coadministered immunosuppressive agents.^116^ Ursodeoxycholic acid was recently introduced for NASH management in clinical practice. However, findings on its actual influence on liver histology remain inconclusive.^117^ Research on the role of metformin, obeticholic acid, n‐3 polyunsaturated fatty acids, pentoxifylline, and orlistat has produced inconclusive results.^97^, ^103^, ^114^

A meta‐analysis by Saab et al. confirmed a previously held statement that caffeine consumption reduces the risk of NAFLD and also improves fibrosis in patients with NASH.^118^

Much research has been performed to develop effective and safe pharmacological treatments for NAFLD. However, to date, dietary restrictions and lifestyle modifications remain the only universally accepted therapeutic options. NAFLD in the posttransplant setting may result from de novo development of the disease or its recurrence in the transplanted organ and adversely affects concomitant metabolic derangements. Furthermore, in addition to obesity and DM2, NAFLD is considered as a risk factor for HCC development and it is related with multiple comorbidities. Notably, NAFLD may occur in individuals with normal body weight and serum transaminase levels within the reference range. Currently, no transplant‐specific guidelines are available for NAFLD screening or management, and recommendations applicable to the general population are used in this specific patient group.

## 5 MICROBIOME

Over the course of recent years, gut microbiota has attracted significant attention as a prospective therapeutic target for metabolic disorders. Emerging scientific evidence confirms the multidirectional influence of intestinal bacteria on human metabolism, liver steatosis, and maintenance of the intestinal epithelium barrier integrity, which is known to be disrupted in many chronic diseases, such as obesity. The intestinal microbiome may affect energy balance promoting extra energy harvest from the diet and influencing its further utilization and storage, all of which lead to increased body fat content, triglyceride accumulation in the liver, and insulin resistance.^119^, ^120^ A growing body of evidence postulates the potential causative role of intestinal microbiota in DM2, lipid disorders, MS, NAFLD, HCC, and even CVDs.^120^, ^121^, ^122^, ^123^ Short‐chain fatty acids (SCFAs), major end‐products of the bacterial fermentation process, may provide up to 10% of the human daily energy requirement and produce multidirectional effects on gastrointestinal tract function.^124^ SCFAs are a source of energy for the intestinal epithelium cells and hepatocytes, they possess antiinflammatory properties, reduce intestinal permeability and regulate energy homeostasis by acting on G protein receptors stimulating the release of molecular particles responsible for controlling appetite, insulin release, and gastrointestinal tract function: glucose‐dependent insulinotropic polypeptide, glucagon‐like peptide‐1, and YY peptide.^125^, ^126^, ^127^, ^128^ Additionally, liver transplant recipients' exposure to numerous factors that predict intestinal microbiota alterations is initiated in the pretransplantation period and continues life‐long.

### 5.1 Intestinal microbiota and the liver

The human microbiome comprises seven main bacterial phyla; however, *Bacteroidetes* and *Firmicutes* constitute >90% of the gut microbiota. Dysbiosis shows a well‐documented association with metabolic complications and various diseases of the liver.^129^ Studies on obese animal and human models determined pronounced increase in the *Firmicutes*/*Bacteroidetes* ratio in comparison with lean subjects, which was linked to additional caloric extraction up to 150 kcal daily.^130^ In agreement with this notion, weight loss reduction induced by lifestyle modifications or BS intervention resulted in an increased abundance of *Bacteroidetes* strains and proportional decrease in an abundance of *Firmicutes* strains.^131^, ^132^, ^133^ However, whether alterations in the composition of the intestinal microbiota occur secondary to weight loss or are triggered by dietary changes remains unclear. In contrast, several studies demonstrated no significant pattern between these two dominating bacterial divisions in individuals with and without obesity.^134^ Reduced microbial diversity was the only reproducible outcome observed across most studies. Critical role in mediating obesity development was assigned to *Lactobacillus* and *Bifidobacterium* strains, which belong to *Firmicutes* and *Bacteroidetes* genera, respectively, and are present in commonly available probiotics preparations. Interestingly, results of several studies have confirmed that *Lactobacillus* strains may promote weight gain.^135^, ^136^, ^137^ On the other hand, one of the first published studies that analyzed alterations in the composition of the human intestinal microbiome at the species level, rather than the genus level, indicates a significantly more complex interplay between obesity and the gut microbiome documenting obesity association with lower levels of *Bifidobacterium* animalis, *Lactobacillus paracasei*, and *Lactobacillus plantarum*, and higher levels of *Lactobacillus reuteri*.^138^, ^139^, ^140^ Accordingly, *L. reuteri* is used in agriculture to ensure healthy growth and weight gain in livestock.^135^ In similarity to the obesity‐related findings, increased abundance of *Lactobacillus* strains was also reported in patients with NAFLD compared with healthy controls, although a recent meta‐analysis reported no differences in the abundance of *Bacteroidetes* or *Firmicutes* between patients with NAFLD and controls.^141^, ^142^ Research on animal models of NASH determined significant alterations in intestinal bacterial diversity, with a distinct imbalance between probiotic and pathogenic bacterial strains resulting in disease progression.^129^, ^143^, ^144^ Gut dysbiosis in cirrhotic patients is a proven phenomenon participating in disease progression and affecting morbidity and mortality rates.^129^, ^145^ There is a paucity of scientific information on gut microbiota alterations following liver transplantation. However, emerging evidence suggests decreased microbiome diversity, increased abundance of pathogenic bacterial strains, and decreased abundance of butyrate‐producing bacteria in liver transplant recipients compared with healthy controls.^146^, ^147^ Interestingly, both compositional and functional alterations in the intestinal microbiota show partial improvement between the 12th and 24th month following liver transplant.^147^

Divergent outcomes of studies may suggest a significantly more complex interplay between obesity/NAFLD and gut microbiome alterations, which may not be limited to evaluation of a single parameter or may equally be a result of different methodology adopted in the studies. Furthermore, significant heterogeneity of the available research and individual variability in the investigated cohorts may be relevant in this context. Further studies are warranted to comprehensively elucidate the complex interplay between the intestinal microbiome ecosystem and metabolic derangement in the host.

### 5.2 Probiotics and prebiotics

Studies that have investigated gut microbiome modifications have shown promising results; the administration of certain probiotic bacteria, particularly *Lactobacillus* and *Bifidobacterium*, was associated with beneficial effects on the metabolic profile. *Bifidobacterium* supplementation promoted weight loss, reduced intestinal inflammation, and improved intestinal barrier integrity.^147,148^ *Lactobacillus* strains reduced the amount of visceral adipose tissue and the size of adipocytes.^149^ Moreover, administration of probiotics, specifically *Lactobacillus* strains, was associated with multidirectional beneficial effects with regard to infection incidence and graft rejections episodes, mortality, and length of hospitalization in the posttransplant setting.^149^ Prebiotics administration in animal models led to increased abundance of *Bifidobacterium* and *Lactobacill*us strains and improved the carbohydrate parameters of the metabolic profile.^150^ RCTs in humans replicated these findings.^151^

Mounting evidence has indicated the role of probiotics/symbiotics as potential therapeutic targets in NAFLD management, which reduce liver steatosis and inflammation. However, despite the promising short‐term results, further studies are required to conclusively establish the long‐term safety and efficacy of their administration.^152^, ^153^ Although auspicious metabolic outcomes are mainly extrapolated from the general population studies or from animal research, some studies have hypothesized that gut microbiota modification may serve as a future therapeutic target for prophylaxis or against recurrence of NAFLD and even HCC development following liver transplant.^143^, ^154^ Intriguingly, studies conducted on animal models suggest that probiotic administration may be helpful in mitigating the noxious effects of immunosuppressive agents.^155^

### 5.3 Fecal microbiota transplantation

FMT methods are currently experiencing their renaissance. A randomized double‐blind pilot study that investigated the FMT procedure in patients with MS confirmed substantial improvement in peripheral insulin sensitivity, 6 weeks postprocedure. Observed alterations in gut microbiota suggest a significant influence of butyrate‐producing commensal bacteria on the study outcomes.^156^ A recent meta‐analysis by Proença et al. supports the overall safety of FMT as gut microbiome‐targeted therapy. Regretfully, the efficacy of the procedure renders to be poorly substantiated.^157^ Yet to be answered is a question about the potential risk of FMT in immunocompromised populations. Most of the reported undesirable effects were mild. However, the study by Kelly et al. demonstrated that immunocompromised individuals' response to FMT may not be as beneficial and safe as in the general population documenting severe adverse events in 15% of study participants, with some of them requiring hospitalization.^158^ No literature findings reported procedure‐related spread of transmissible disease, albeit such threat has not been ruled out completely.

Based on currently available data, mostly originating from general population studies, prebiotics may be safely administered in the transplant population. Probiotics have shown promising results with regard to microbiome modifications, hence, may serve as potential therapeutic agents for obesity and NASH management. FMT is an intriguing subject of scientific research; however, several safety‐ and efficacy‐related questions remain to be answered with regard to immunocompromised individuals.

## 6 OBESITY AND DRUG METABOLISM

Obesity, as a chronic condition associated with low‐grade inflammation, affects gut permeability, gastric emptying, cardiac output, and liver blood flow, and is suggested to promote alterations in the PK and/or pharmacodynamic (PD) properties of administered medicines. Excessive fat accumulation together with chronic inflammation may exercise a considerable impact on activity of hepatic enzymes, as well as on expression of hepatic drug transporters. In the latter case, immunosuppressive treatment appears to be an additional agent of influence. Of note, advancement and duration of obesity may play a role in this process.^159^ Nevertheless, interplay between obesity and drug metabolism appears to be complex and multifactorial, with high individual variability.

### 6.1 Obesity and cytochrome enzymes activity

Cytochrome CYP3A4 is known to mediate approximately 50% of the Phase 1 reactions of drug metabolism.^159^ Importantly, key immunosuppressive agents such as cyclosporine, tacrolimus, sirolimus, and MPA undergo extensive biotransformation via this metabolic pathway.^160^ Several studies have documented reduced CYP3A4 activity in patients with obesity and NAFLD, albeit, the clinical relevance of these findings is not known.^159^, ^161^ However, this fact may play a vital role in the metabolism of drugs with a narrow therapeutic range, when slight fluctuation in their serum concentrations may produce significant therapeutic consequences and result in ineffective treatment or drug‐related toxicity. Of note, CYP3A4 activity showed a tendency to normalize following weight‐reduction surgeries, regardless of the surgical technique, which concurs with the results of studies performed in patients after liver transplantation, in whom higher doses of immunosuppressants were required after BS.^159^, ^161^, ^162^ However, the anatomical and physiological implications of surgical interference in the gastrointestinal tract may provide an alternative justification for these findings.^160^ Currently, the question about the required dose adjustment of CYP3A4 substrates in individuals with obesity following BS remains unanswered. Further studies are warranted for comprehensive evaluation of BMI and surgically induced weight loss correlation with cytochrome P450 (CYP) enzyme activity to draw definitive conclusions and lay the groundwork for issuing possible recommendations. Moreover, obesity was demonstrated to increase cytochrome CYP2E1 activity, as well as Phase 2 of metabolic reactions.^159^, ^161^ While enzymatic activity of CYP2E1 showed a positive correlation with total body weight and NAFLD, an inverse association was observed with an increasing degree of hepatic steatosis.^159^, ^161^, ^163^

### 6.2 Drug interactions

Pursuant to currently effective recommendations, 3‐hydroxy‐3‐methylglutaryl coenzyme reductase inhibitors (statins) are the drugs of choice for the pharmacological management of hyperlipidemia in the posttransplant setting.^164^ However, combined therapy with statins and calcineurin inhibitors (CNIs) may result in clinically relevant drug–drug interactions, which increase the risk of adverse events. Since both tacrolimus and cyclosporine are inhibitors of cytochrome CYP3A4, concomitant administration of CNIs and statins may theoretically increase plasma statin levels. However, no clinically relevant inhibition was reported in vivo for tacrolimus.^165^ Therefore, it is generally advised to prefer fluvastatin or pravastatin preparations in patients with cyclosporine‐based immunosuppressive treatment as fluvastatin is primarily metabolized by CYP2C9 and metabolism of the latter is predominantly cytochrome‐independent.^164^

Based on the current scientific findings, a hypothesis of drug interactions between CNIs and statins based solely on a common metabolism path via cytochromes appear to be an oversimplification since a few studies have reported cases of cyclosporine‐induced excessive systemic exposure to statins not metabolized by cytochrome CYP3A4.^165^ A recent study on CNIs metabolism indicates an equally important role of statin membrane transporting P‐glycoprotein (P‐gp) and organic anion transporting polypeptides (OATPs) in the PK and PD properties as they are known to mediate medicine disposition.^165^, ^166^ The OATP1B1 subtype, which shows expression mainly on the basolateral hepatocytes surface, may be especially important in mediating toxic effects of statins administration. Moreover, the study highlights the substantial differences in the PK properties of cyclosporine and tacrolimus. Cyclosporine appeared to be a strong inhibitor of OATP1B1, which facilitates hepatic uptake of statins, whereas tacrolimus showed a negligible effect.^165^ Furthermore, cyclosporine has been found to significantly affect P‐gp activity reducing it even by 50%.^165^ Interestingly, emerging data originating from studies in animal models have reported a significant association between NAFLD and reduced expression of other hepatic drug transporters, which was shown to be reliant on disease progression.^167^ Research in humans, concededly restricted, is in line with these statements.^168^ These findings are speculated to mediate drug‐induced toxicity.

### 6.3 Obesity and antimicrobial treatment

Obesity should be taken under advisement during antimicrobial treatment, particularly in patients in whom therapy produces unsatisfactory results; several studies have reported that compared with control groups, patients with obesity show reduced tissue penetration of some antibiotics. The best relationship was documented with cefazolin and ciprofloxacin.^159^, ^161^, ^169^, ^170^ These findings may suggest that an increased dosage or frequency of antibiotic administration should be considered in patients with obesity, before switching to alternative antimicrobial regimens, regardless of the elevated plasma levels of the medication.^171^, ^172^

Numerous studies have shown that both obesity and NAFLD may significantly affect the activity of hepatic enzymes and hepatic drug transporter expression and thereby affect the PK and/or PD properties of administered medicines. Compared with tacrolimus‐based regimens, cyclosporine‐based immunosuppressive regimens tend to predispose patients to potential drug–drug interactions.

## 7 IMMUNOSUPPRESSION

Numerous studies recommend immunosuppressive treatment without or with prompt cessation of glucocorticosteroids in patients with obesity, ergo with high CV risk.^164^, ^173^, ^174^ This approach is expected to curb weight gain and decrease the incidence or preclude exacerbations of metabolic complications in the posttransplant period. To date, the long‐term outcomes of such immunosuppressive schedules are not well investigated.

### 7.1 General recommendations

According to the latest guidelines issued by the International Liver Transplantation Society, corticosteroid therapy cessation is recommended by the end of the first 3 months following transplantation with subsequent CNIs monotherapy. Patients at a high immunological risk may be candidates for long‐term corticosteroids administration at low doses or may be eligible for steroid replacement therapy, where antiproliferative agents are substituted for an oral steroid.^164^ Several publications describe that in addition to corticosteroid avoidance, tapering the CNIs dosage appears to be an important step to reduce metabolic complications, as numerous metabolic adverse effects of glucocorticosteroids, such as arterial hypertension, lipid metabolism, or glucose tolerance disorders, are also associated with CNIs therapy.^20^, ^164^, ^173^, ^174^ These deleterious effects of CNIs preparations are proposed to arise from their vasoconstrictor activity, inhibition of prostacyclin and nitric oxide production, increased release of thromboxane and endothelin, and increased sodium and water reabsorption.^175^ Therefore, dual therapy with CNIs and mycophenolate mofetil (MMF) or mammalian target of rapamycin inhibitors (mTORis) may serve as an alterative that enables CNIs dose reduction mitigating drug‐related metabolic risk and toxicity.^164^, ^173^, ^176^ Table 3.

**Table 3:** The impact of immunosuppressive drugs on metabolic complications

	Obesity	Diabetes mellitus	Hyperlipidemia	Hypertension
Corticosteroids	+	+++	+	+
Calcineurin inhibitors	+	++	+	++
Mycophenolate mofetil	‐	‐	‐	‐
Mammalian target of rapamycin inhibitors	+	‐	++	+
Thymoglobulin	‐	‐	‐	‐
IL‐2‐receptor antibodies	‐	‐	‐	‐

### 7.2 Role of CNIs

CNI in monotherapy or in combination with steroids at low doses is considered the mainstay of maintenance therapy following liver transplantation. CNIs incorporated into the immunosuppressive regimen significantly improved patient and graft survival rates and reduced the number of acute rejection episodes.^176^ Fussner et al. observed beneficial influence of tacrolimus‐based immunosuppression in reducing CVDs risk.^26^ In contrast, a meta‐analysis by Lan et al. suggested a comparable effectiveness of both CNIs in monotherapy as posttransplantation maintenance therapy, indicating that tacrolimus may be significantly beneficial in patients transplanted for HCV.^174^

### 7.3 Antiproliferative agents

MMF, the preferred immunosuppressive agent among the antiproliferative drugs portfolio, is devoid of nephrotoxic properties and is neutral with regard to metabolic complications development.^164^, ^176^, ^177^

### 7.4 Mammalian target of rapamycin inhibitors

Owing to their pleiotropic antiatherosclerotic properties, mTORis curb weight gain and were shown to lower the risk of CVDs.^173^, ^178^ However, mTORis administration failed to reduce the overall risk of MS development following liver transplant, which could be attributed to the fact that mTORis are not completely free from the metabolic adverse consequences being paradoxically strongly associated with hyperlipidemia.^179^ Despite the documented satisfactory outcomes of mTORis monotherapy, tacrolimus remains an essential component of long‐term posttransplantation therapy.^164^, ^173^, ^176^

### 7.5 Steroids

Steroids are commonly applied as potent agents for prevention and treatment of acute rejection episodes. Therefore, as might be expected, strategy of steroid avoidance or early steroid withdrawal was associated with higher incidence of acute rejection episodes compared with the steroid‐based strategy.^180^ However, as evidenced by several studies, daclizumab induction regimens or CNIs minimization protocols in combination with MMF were shown to reduce metabolic complications, hypertension, and hyperuricemia without unfavorable impact on acute rejection episodes.^177^, ^181^, ^182^ Interestingly, two meta‐analyses showed no significant differences in patients and graft survival, infections rates, and the risk of hypertension between the steroid‐based and steroid‐free group.^180^, ^183^ However, steroids administration was associated with a higher incidence of cytomegalovirus infections, DM, and higher serum cholesterol levels.^183^

Steroid‐free protocols appeared to be particularly favorable for patients transplanted for an HCV indication.^183^, ^184^ Junge et al. in their randomized prospective study reported the beneficial effects of steroid free‐protocols even in patients transplanted for autoimmune hepatitis.^185^ To date, a suitable time frame for steroid withdrawal has not been established in this group of patients. However, based on current data, it may not be feasible or safe earlier than 1 year after the transplant.

Metabolic complications are commonly observed in patients who undergo liver transplantation; therefore, it is challenging to select optimal immunosuppressive regimens that can successfully address all metabolic concerns in these patients. Some medicines, which are beneficial in one metabolic derangement, may adversely affect others. An individualized therapeutic approach is recommended in liver transplant recipients.

## 8 SUMMARY

With the rapidly growing obesity pandemic resulting in a significantly altered profile of patients awaiting liver transplant, it is vitally important to fill the obesity‐related gaps in knowledge and be prepared to confront the changing clinical dilemmas.

Early introduction of dietary and lifestyle education is strongly recommended as MS predominantly occurs between 6 and 12 months following liver transplant, and body weight parameters are known to increase significantly over 6 months posttransplantation.^27^ It is, therefore, reasonable to conclude that in addition to measurement of BMI, WC measurements are important during regular follow‐up to promptly and accurately identify liver transplant recipients at a high risk of metabolic complications.

To date, pharmacological treatment options in obesity or NAFLD have been limited and insufficiently explored to be recommended in everyday practice. Further research is warranted to evaluate the risk‐benefit profile of orlistat before it can be safely incorporated into obesity management regimens in patients after liver transplantation. Most studies reported significant weight loss and improvement in the metabolic profile when orlistat was combined with dietary restrictions and vitamin E. According to the EASL‐EASD‐EASO recommendations, only NASH patients with at least F2 stage of fibrosis may benefit from pioglitazone and short courses of vitamin E. However, currently available data are insufficient and preclude expansion of these findings to all eligible patients as qualification for therapy should be individualized.^97^ BS is deemed a safe alternative for obesity management in liver transplant recipients with morbid obesity, associated with plausible amelioration of other metabolic disorders. Therefore, BS should be considered in eligible individuals to reduce long‐term morbidity and mortality following transplantation.

Quantitative and qualitative alterations in gut microbiota should be taken into account in patients after liver transplant with insufficient or no response to the introduced obesity management plan, especially if they were exposed to repeated or prolonged antimicrobial treatments.

Metabolic complications commonly coexist in patients after liver transplant; therefore, it is vitally important to consciously combine pharmacological treatment. Regular revisions of prescribed and nonprescription medications should be in place to identify possible drug–drug interactions interfering with immunosuppression therapy. Special caution should be applied in the population with obesity as the PK and/or PD properties of medicines may be altered.

The selection of an immunosuppressive regimen that successfully addresses all metabolic concerns, may pose a considerable challenge. Some medicines, which are beneficial in one metabolic derangement, may adversely affect others. Therefore, an individualized therapeutic approach is warranted in liver transplant recipients. An immunosuppressive protocol with a short‐term course of steroid administration followed by early initiation of tacrolimus in monotherapy or steroid replacement therapy combined with MMF appears to be a compelling and acceptable alternative in liver transplant recipients, allowing to achieve optimal results while minimizing immunosuppression‐attributable complications.^181^, ^183^ Special caution is advised in patients with an initially increased risk of acute rejection episodes. Further research is warranted to establish the risk of chronic rejection episodes associated with steroid‐free or steroid early withdrawal regimens.^186^ Based on current knowledge it is a questionable fact if ab initio monotherapy with CNIs should be advised in liver transplant recipients.

Liver transplant patients constitute a specific group among the MS population and require extra caution to achieve optimal therapeutic results with minimization of iatrogenic adverse events. In addition to the well‐known risk factors associated with MS observed in the general population, liver transplant recipients are exposed to the numerous transplant‐specific risk factors such as long‐term immunosuppressive therapy, multiple comorbidities, altered metabolism, or an increase in appetite following liver transplant. Many of these risk factors are nonmodifiable; therefore, it is vitally important to proactively seek and treat the remaining amendable factors. Effective and comprehensive management of metabolic complications is shown to yield multiple beneficial results in the liver transplant population and may bring gratifying results in improving long‐term survival rates.

## AUTHOR CONTRIBUTIONS

*Conceptualization, writing – original draft preparation*: Kinga Czarnecka. *Writing – original draft preparation, visualization*: Paulina Czarnecka. *Conceptualization, writing – review and editing*: Olga Tronina. *Writing – critical review and editing*: Teresa Bączkowska. *Writing – critical review and editing*: Magdalena Durlik. All authors have substantially contributed to conducting the underlying research and drafting this manuscript.

## CONFLICT OF INTERESTS

The authors have no conflicts of interest to declare.
